# Assembly properties of bacterial tubulin homolog FtsZ regulated by the positive regulator protein ZipA and ZapA from *Pseudomonas aeruginosa*

**DOI:** 10.1038/s41598-020-78431-x

**Published:** 2020-12-07

**Authors:** Mujeeb ur Rahman, Zhe Li, Tingting Zhang, Shuheng Du, Xueqin Ma, Ping Wang, Yaodong Chen

**Affiliations:** 1 Key Laboratory of Resources Biology and Biotechnology in Western China, Ministry of Education, College of Life Sciences, Northwest University, Xi’an, 710069 Shaanxi China; 2 Department of Anesthesiology, Duke University Medical Center, Durham, NC 27710 USA

**Keywords:** Biochemistry, Biophysics, Microbiology

## Abstract

Bacterial tubulin homolog FtsZ self-assembles into dynamic protofilaments, which forms the scaffold for the contractile ring (Z-ring) to achieve bacterial cell division. Here, we study the biochemical properties of FtsZ from *Pseudomonas aeruginosa* (PaFtsZ) and the effects of its two positive regulator proteins, ZipA and ZapA. Similar to *Escherichia coli* FtsZ, PaFtsZ had a strong GTPase activity, ~ 7.8 GTP min^-1^ FtsZ^-1^ at pH 7.5, and assembled into mainly short single filaments in vitro. However, PaFtsZ protofilaments were mixtures of straight and “intermediate-curved” (100–300 nm diameter) in pH 7.5 solution and formed some bundles in pH 6.5 solution. The effects of ZipA on PaFtsZ assembly varied with pH. In pH 6.5 buffer ZipA induced PaFtsZ to form large bundles. In pH 7.5 buffer PaFtsZ-ZipA protofilaments were not bundled, but ZipA enhanced PaFtsZ assembly and promoted more curved filaments. Comparable to ZapA from other bacterial species, ZapA from *P. aeruginosa* induced PaFtsZ protofilaments to associate into long straight loose bundles and/or sheets at both pH 6.5 and pH 7.5, which had little effect on the GTPase activity of PaFtsZ. These results provide us further information that ZipA functions as an enhancer of FtsZ curved filaments, while ZapA works as a stabilizer of FtsZ straight filaments.

## Introduction

Prokaryotic cell division is mediated by a protein complex known as the divisome^1–5^. Assembly of this machinery is initiated by the polymerization of FtsZ, a bacterial tubulin homolog, into a contractile ring (Z-ring), at the future division site^6^. FtsZ protofilaments (pfs), attached onto the inner membrane by other membrane proteins, function as a scaffold for the recruitment of downstream division proteins to develop the Z-ring, as well as generate an inward force to initiate the bacterial cell division^7,8^.

The FtsZ from *Escherichia coli* (EcFtsZ) is well investigated in many studies. EcFtsZ self-assembles into mostly single dynamic pfs in the presence of GTP in vitro, averaging a length of approximately 200 nm^9,10^. Fluorescence studies showed that EcFtsZ filaments assembled with rapid nucleated kinetics upon addition of GTP, and at steady state had a very fast subunit exchange rate of 5–7 s^10,11^. This in vitro exchange is accordant with the 8–10 s half-time for FtsZ subunits turnover in the Z-ring in vivo, measured by FRAP (fluorescence recovery after photobleaching) technique^12,13^. Recent studies using super-resolution fluorescence microscopy discovered that FtsZ polymer patches move around the circumference of the cell by treadmilling and guide the new cell wall synthesis in vivo^14–18^. Treadmilling FtsZ has also been observed and characterized in vitro, with FtsZ pfs attached to the lipid membrane^19,20^.

In *E. coli*, the Z-ring contains more than 20 associated proteins, including both negative and positive regulators^1,3,21^. The negative regulators, such as MinC and SulA, could inhibit FtsZ polymerization directly and regulate the Z-ring formation^22–26^. The positive regulators, including ZipA (FtsZ interacting protein A) and ZapA (FtsZ associated protein A), may enhance FtsZ assembly in vitro, promote their lateral contact to form bundles, and/or stabilize the Z-ring structure in vivo^27–29^. The primary assembly of the first protein complex, the proto-ring, involves the interaction of FtsZ with two other proteins, ZipA and FtsA, which tether and stabilize the Z-ring onto the inner membrane^30–34^. Although nonessential for divisome formation, the Zap family proteins are considered as regulation components of the proto-ring to stabilize the Z-ring. In *Bacillus subitilis*, since there is no ZipA protein, it is reported that divisome proteins FtsA, SepF, ZapA and EzrA are involved in the early proto-ring assembly^35^.

Both ZapA and ZipA are considered as positive regulators of the Z-ring. ZapA, is a small cytoplasmic protein, broadly conserved protein among bacteria, that enhances the stability of the FtsZ ring^29^. ZapA forms dimers and tetramers in vitro and was reported to promote FtsZ bundles by cross-linking adjacent FtsZ pfs^36–38^. A ZapA deficient strain in vivo shows no phenotype under normal condition but displays severe division impairment if FtsZ expression level is low, or the ezrA gene is knocked out^29^. Even though ZapA promotes FtsZ bundling, it is reported that ZapA had only a mild effect on the GTPase activity of FtsZ^29,37^, and had little effect on the treadmilling activity of FtsZ filaments^20^.

ZipA, a transmembrane protein, exists only in γ-proteobacteria, including *E. coli* and *Pseudomonas aeruginosa*, with a cytoplasmic domain that binds the C-terminal peptide of FtsZ^30^. ZipA’s function in vivo is still controversial. Previous studies suggested that ZipA has the functions of not only attaching FtsZ filaments onto membrane, but also stabilizing the Z-ring by cross-linking adjacent FtsZ pfs to form FtsZ bundles^28,39^. However, recent studies challenged that since ZipA from *E. coli* only induced FtsZ bundling if the pH is below 7^19,20,40,41^. At the physiological pH 7.5, FtsZ, together with ZipA, still assembles into single filaments. Furthermore, our previous results suggested that ZipA enhances and stabilizes the highly curved FtsZ-GDP filaments; this function might be coupled with the constriction forces generation^41^. ZipA is an essential protein for cell division under the normal condition^27^; however, some FtsA mutants from *E. coli* may bypass the need of ZipA^42,43^. A suggested mechanism is that these mutants might reduce the FtsA self-interaction and thus enhance the recruitment of downstream proteins^44^. Interestingly, our previous results found the FtsA* (one of FtsA mutants that bypassed the need of ZipA) also stabilized the FtsZ-GDP highly curved conformation^41^, partially overlapping with the function of ZipA. In order to understand whether this effect is universal, in the present study we examined the effects of ZipA from *P. aeruginosa* on FtsZ polymerization. ZapA is another positive regulated proteins of FtsZ filaments. It is also interesting to know whether ZapA contributes to the bending confirmation or not.

We investigated here the assembly properties of FtsZ from *P. aeruginosa* (PaFtsZ) and the effects of ZipA and ZapA in vitro. We found that PaFtsZ pfs are mixtures of straight and curved. ZapA from *P. aeruginosa* (PaZapA) enhances FtsZ bundling, similar to other bacterial species. Meanwhile, ZipA from *P. aeruginosa* (PaZipA) only promotes FtsZ bundling when the pH is 6.5. At the physiological pH 7.5, PaZipA mostly promotes the formation of single and curved FtsZ filaments, which include not only FtsZ-GDP highly curved conformation, but also the FtsZ-GTP intermediate curved conformation. it is consistent with our previous results from *E. coli*^41^. It confirms that ZipA may contribute by stabilizing the bending conformation and enhancing the constriction forces during cytokinesis.

## Results

### PaFtsZ assembles into both straight and curved filaments

**Figure 1:**
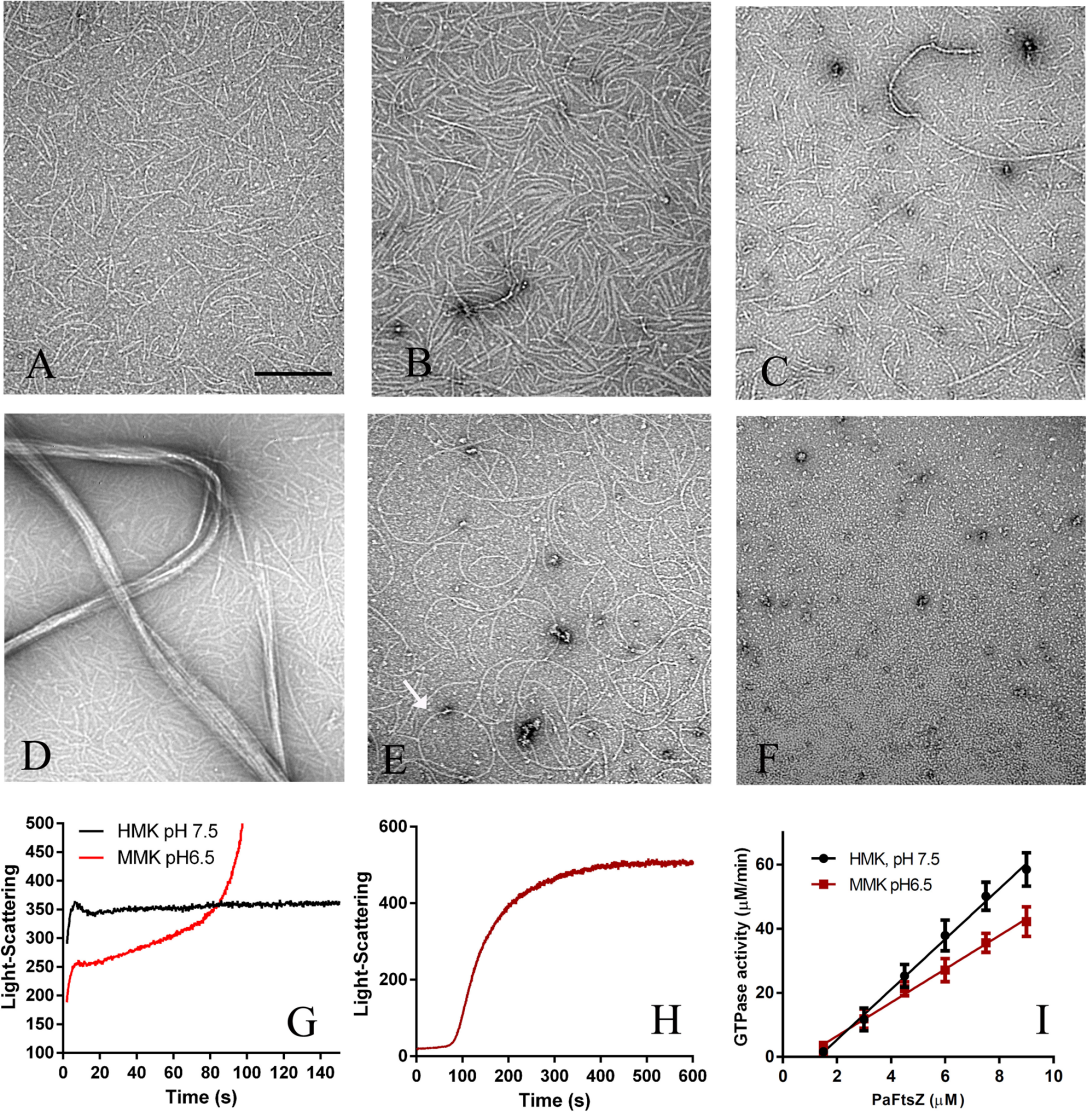
**(A–F)** Negative stain EM of PaFtsZ assembly in different buffer conditions. **(A,B)** 3 and 5 µM PaFtsZ in HMK (50 mM HEPES, 100 mM KAc, 5 mM MgAc, pH 7.5). **(C,D)** 5 µM PaFtsZ in MMK (50 mM MES, 100 mM KAc, 5 mM MgAc, pH 6.5) at 1 min **(C)** and 5 min after adding GTP **(D)**. **(E)** 5 µM PaFtsZ in MEK (50 mM MES, 100 mM KAc, 1 mM EDTA, pH 6.5). The arrow indicates a close circle. **(F)** 10 µM PaFtsZ in HEK (50 mM HEPES, 100 mM KAc, 1 mM EDTA, pH 7.5). The bar equals 200 nm and all EM images have the same magnification. **(G,H)** Assembly kinetics of 5 µM PaFtsZ measured by the light-scattering assay. PaFtsZ assembled mostly single filaments within 10 s in HMK buffer **(G)**, but in MMK buffer, it is a two-stage assembly: single filaments assembled within 10 s, followed by a second increase in light scattering attributed to bundling **(H)**. Assembly in MMK followed for a longer time and on a larger light scattering scale **(H)**. It is worth mentioning that the intensity values between **(G)** and **(H)** are not comparable. We reduce the sensitivity of the detector to measure the bundling formation **(H)**. **(I)** GTPase activity of PaFtsZ at different concentrations in HMK and MMK buffer. GTP is 0.5 mM.

Although PaFtsZ has been used in some studies, its detailed biochemical characteristics are still not fully reported. Here, we first examined its assembly using both negative-stain electron microscopy (EM) and light-scattering assays. In these studies, we used several different buffers. HMK buffer contains 50 mM HEPES, pH 7.5, 100 mM KAc and 5 mM MgAc. The pH value of HMK buffer is close to the physiological condition. MMK buffer contains 50 mM MES, pH 6.5, 100 mM KAc and 5 mM MgAc. Usually, EcFtsZ assembly is better in MMK buffer and is referred to as the assembly buffer. HEK buffer and MEK buffer use 1 mM EDTA to replace MgAc of HMK and MMK buffers; FtsZ assembly still occurs in the absence of Mg, but GTP hydrolysis is completely blocked^9,45^. Similar to EcFtsZ, PaFtsZ assembles into single pfs in HMK buffer (Fig. 1A,B). Most PaFtsZ pfs are straight but some are arc-shaped, with a 100–200 nm diameter characterized as “intermediate curvature”^5^. The intermediate curved conformation was enhanced when PaFtsZ assembled in MEK buffer, which contains no Mg^2+^ at pH 6.5 (Fig. 1E). Occasionally, PaFtsZ formed closed circles in MEK buffer (Fig. 1E, arrowed). Assembly in HEK buffer (pH 7.5) was much weaker than in MEK (pH 6.5), producing few pfs even at 10 µM PaFtsZ (Fig. 1F).

In HMK buffer the assembly gave mostly single pfs at 3 µM PaFtsZ, while at 5 µM these tended to form thin bundles, as indicated by the higher contrast (Fig. 1A,B). In MMK buffer assembly produced mostly single pfs after 1 min, but these aggregated into thick bundles by 5 min (Fig. 1C,D). Light scattering confirmed the bundling and showed that assembly in MMK followed two stages. Assembly of single pfs gave an initial rise in light scattering at ~ 10 s both in HMK and MMK buffer (Fig. 1G), which is similar to the assembly of EcFtsZ detected by fluorescence assays^9,10^. This was followed after ~ 60 s by a large increase in light scattering attributed to bundling in MMK buffer (Fig. 1G,H). To observe this second stage more fully we reduced the slit width of the spectrofluorometer. In Fig. 1H the initial formation of single pfs is hidden in the lag phase, but after ~ 100 s light scattering increases strongly due to bundling.

PaFtsZ has a strong GTPase activity, similar to EcFtsZ. In our measurements, the GTPase activity of PaFtsZ in HMK buffer is around 7.8 ± 0.2 GTP min^-1^ FtsZ^-1^ with a 1.3 µM critical concentration (Cc) and around 5.2 ± 0.2 GTP min^-1^ FtsZ^-1^ with a Cc of 0.8 µM in MMK buffer at room temperature (Fig. 1I, Table 1). It is a little stronger than EcFtsZ.

**Table 1:** GTPase activity of PaFtsZ in the absence of or in the presence of ZipA or ZapA in MMK and HMK buffer.

Samples	HMK buffer (pH 7.5)^a^		MMK buffer (pH 6.5)^b^	
	GTPase activity (GTP min^-1^ FtsZ^-1^)	Cc (µM)	GTPase activity (GTP min^-1^ FtsZ^-1^)	Cc (µM)
PaFtsZ	7.8 ± 0.2	1.3 ± 0.2	5.2 ± 0.2	0.8 ± 0.2
PaFtsZ + 5 µM PaZipA	6.9 ± 0.5	0.8 ± 0.4	4.1 ± 0.4	1.0 ± 0.4
PaFtsZ + 10 µM PaZipA	6.5 ± 0.1	1.0 ± 0.1	3.2 ± 0.1	1.4 ± 0.2
PaFtsZ + 5 µM PaZapA	6.7 ± 0.2	0.8 ± 0.2	5.0 ± 0.4	0.7 ± 0.4
PaFtsZ + 10 µM PaZapA	6.5 ± 0.4	0.8 ± 0.3	5.9 ± 0.4	1.2 ± 0.3

*Cc* critical concentration.

^a^HMK buffer: 50 mM HEPES, 100 mM KAc, 5 mM MgAc, pH 7.5.

^b^MMK buffer: 50 mM MES, 100 mM KAc, 5 mM MgAc, pH 6.5.

### PaZipA enhances PaFtsZ assembly and stabilizes PaFtsZ curved conformation in GTP

**Figure 2:**
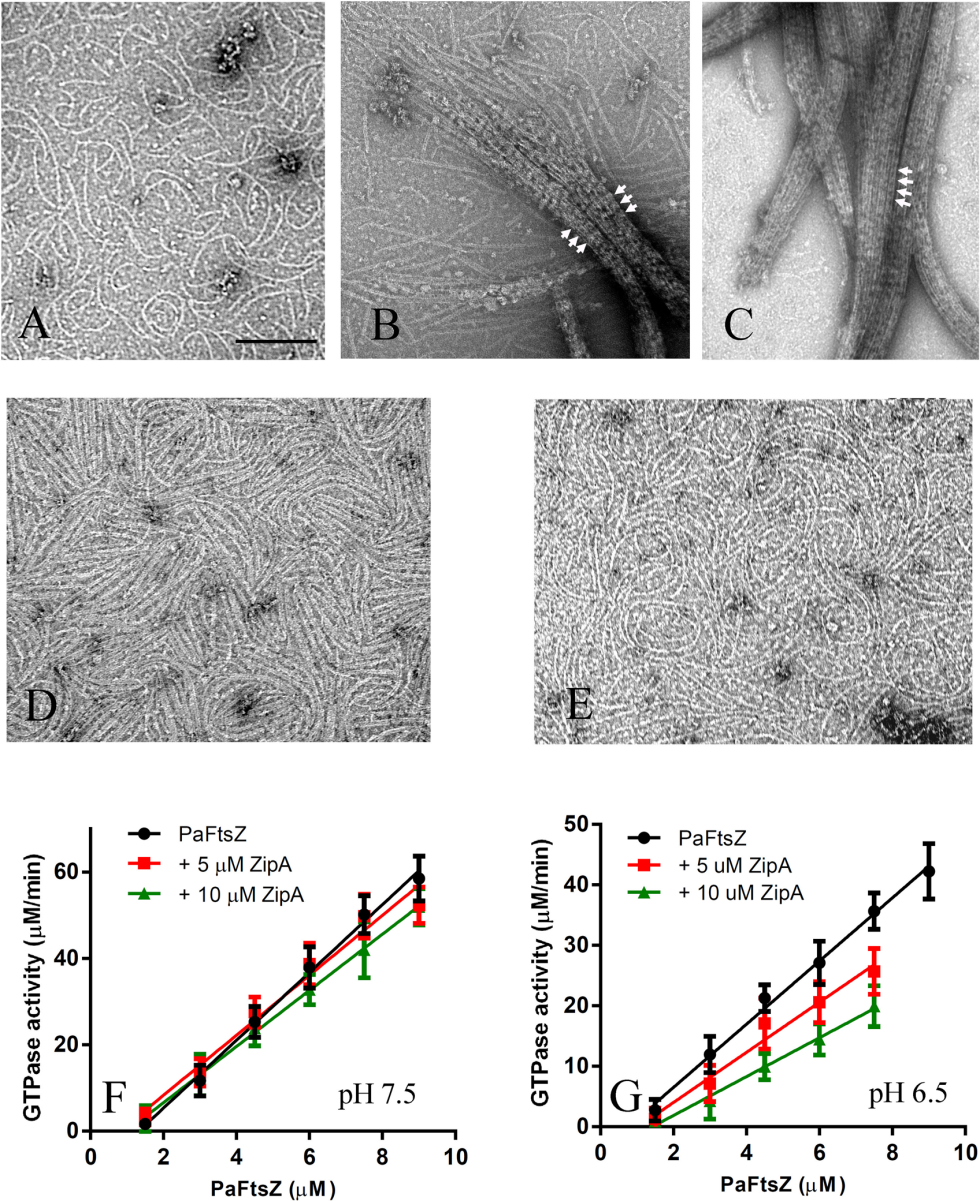
PaFtsZ assembly in the presence of ZipA. **(A–E)** shows negative stain EM of 5 µM PaFtsZ and 10 µM ZipA in HMK buffer **(A),** in MMK buffer **(B,C)**, in MEK buffer **(D)** and in HEK buffer **(E)**. PaFtsZ-ZipA assembled into the huge bundles in MMK buffer; periodic striations (arrows) may be ZipA crosslinks. The bar represents 200 nm and all EM images have the same magnification. **(F–G)** shows the GTPase activity of PaFtsZ in the presence of ZipA in HMK buffer **(F)** and MMK buffer **(G)**.

ZipA only exists in γ-proteobacteria. The identity of ZipA protein between *E. coli* and *P. aeruginosa* is around 30% (Figure S1A). In general, ZipA is considered to be a Z-ring stabilizer based on early studies showing that ZipA from *E. coli* induced the FtsZ filament bundling^28,39^. However, in our recent study, we demonstrated that ZipA effects on EcFtsZ assembly varied with pH and may function as curved filaments enhancer, not Z-ring stabilizer ^41^. To this end, we investigated the effects of PaZipA. PaZipA used in this study is an N-terminally truncated to remove the transmembrane domain for better solubility. Here we report similar results for PaZipA. PaZipA induces PaFtsZ bundling formation in MMK buffer at pH 6.5 (Fig. 2B,C). However, in HMK buffer (pH 7.5), which is close to the physiological condition, PaFtsZ pfs are still single, but more curved (Fig. 2A) than that of PaFtsZ alone (Fig. 1A,B). The diameter of PaFtsZ curved filaments assembled with PaZipA is around 50–100 nm. It is worth noting that PaFtsZ–PaZipA mixture could also assemble into curved filaments in MEK (Fig. 2D) and HEK buffer (Fig. 2E); PaFtsZ alone could not assemble in HEK buffer (Fig. 1E). This indicates that PaZipA can both enhance PaFtsZ assembly and promote PaFtsZ curved conformation.

Similar to previous studies on ZipA from *E. coli*, PaFtsZ–PaZipA assembled into huge, tight bundles in pH 6.5 solution (Fig. 2B,C). We observed the single or double PaFtsZ filaments form tight bundles through the lateral contacts, cross-linked by most likely PaZipA proteins in MMK buffer (Fig. 2B,C, arrowed). In MEK buffer, the addition of PaZipA to PaFtsZ greatly enhanced assembly, producing thick filaments and bundles (Fig. 2D).

Next, we checked the GTPase activity of PaFtsZ in the presence of PaZipA. In HMK buffer the GTPase activity of PaFtsZ decreased slightly: from 7.8 ± 0.2 GTP min^-1^ FtsZ^-1^ without PaZipA to 6.9 ± 0.5 GTP min^-1^ FtsZ^-1^ with 5 µM PaZipA, and to 6.5 ± 0.1 GTP min^-1^ FtsZ^-1^ with 10 µM PaZipA (Fig. 2F, Table 1). In MMK buffer, the GTPase activity of PaFtsZ was reduced much more: from 5.2 ± 0.2 GTP min^-1^ FtsZ^-1^ without PaZipA, to 4.1 ± 0.4 GTP min^-1^ FtsZ^-1^ with 5 µM PaZipA, and 3.2 ± 0.1 GTP min^-1^ FtsZ^-1^ with 10 µM PaZipA, a 40% reduction (Fig. 2G, Table 1). The reduced GTPase is consistent with the huge bundling formation in MMK buffer.

### PaZapA induced PaFtsZ to assemble into large straight sheets and bundles

**Figure 3:**
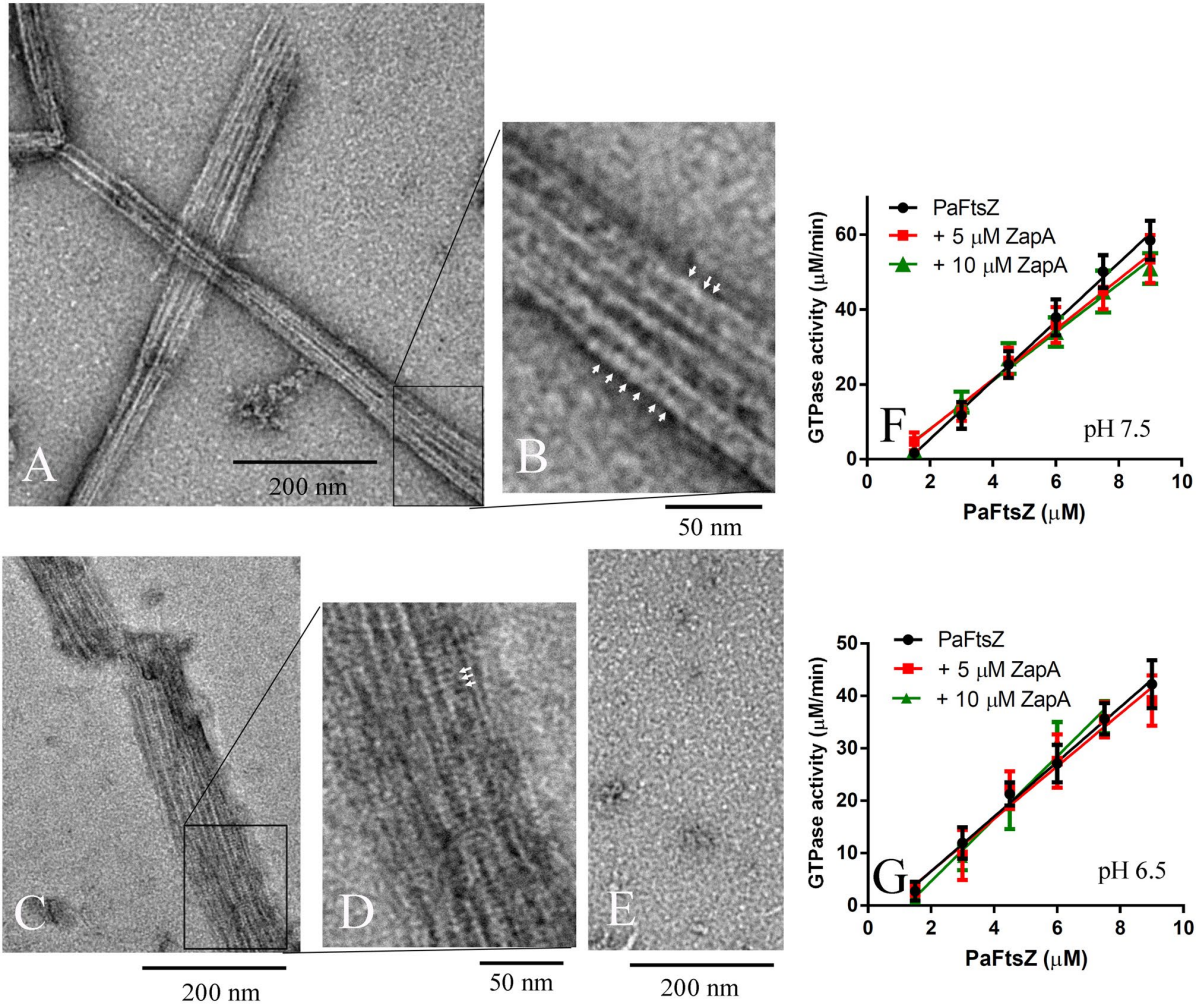
PaFtsZ assembly in the presence of ZapA. **(A–E)** shows negative stain EM of 5 µM PaFtsZ and 10 µM ZapA in MMK buffer **(A,B)**, MEK buffer **(C,D)**, and HEK buffer **(E)**. The arrows in **(B,D)** show periodic striations that be due to ZapA crosslinks. **(F,G)** show the GTPase activity of PaFtsZ with ZapA in HMK buffer **(F)** and MMK buffer **(G)**.

Unlike PaZipA, PaZapA induced PaFtsZ to form large straight sheets and bundles in most buffers we used, including HMK, MMK and MEK buffer (Fig. 3A–D). No assembly was observed in HEK buffer (Fig. 3E), similar to PaFtsZ alone, but different from PaFtsZ with PaZipA. Apparently, PaZapA induced PaFtsZ to form loose bundling structures, and the distance between each PaFtsZ filament is relatively large, from 5 to 11 nm. We also observed some striations across the bundles, which might be ZapA cross-links (Fig. 3B,D, arrowed). The distances between ZapA molecules are different in Fig. 3B,D. It is around 3.5 nm in Fig. 3D, which suggests that ZipA binds to each FtsZ molecule. Meanwhile, it is about 7.9 nm in Fig. 3B, which indicates that every two FtsZ molecules have a ZapA binding.

Although PaFtsZ–PaZapA assembled into large bundles, this had only minor effects on the PaFtsZ GTPase activity both in HMK (Fig. 3F) and MMK buffers (Fig. 3G). In HMK buffer, the GTPase activity is around 7.8 ± 0.2 GTP min^-1^ FtsZ^-1^ without PaZapA, 6.7 ± 0.2 GTP min^-1^ FtsZ^-1^ with 5 µM PaZapA, and 6.5 ± 0.4 GTP min^-1^ FtsZ^-1^ with 10 µM PaZapA (Fig. 3F, Table 1). In MMK buffer, the GTPase is around 5.2 ± 0.2 GTP min^-1^ FtsZ^-1^ without PaZapA, 5.0 ± 0.4 GTP min^-1^ FtsZ^-1^ with 5 µM PaZapA, and 5.9 ± 0.4 GTP min^-1^ FtsZ^-1^ with 10 µM PaZapA (Fig. 3G, Table 1).

### Assembly kinetics of PaFtsZ are modulated by PaZipA or PaZapA

**Figure 4:**
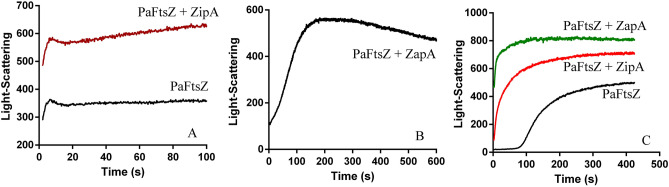
Kinetics of PaFtsZ assembly in the absence of or in the presence of ZipA or ZapA followed by light scattering. **(A)** shows the pfs formation of 5 µM PaFtsZ with or without 10 µM PaZipA in HMK buffer. **(B)** shows the bundles formation of 5 µM PaFtsZ with 10 µM PaZapA in HMK buffer measured by low sensitive light scattering. **(C)** shows the bundles formation of 5 µM PaFtsZ, 5 µM PaFtsZ with 10 µM PaZipA, and 5 µM PaFtsZ with 10 µM PaZapA in MMK buffer.

We compared the assembly kinetics of PaFtsZ, PaFtsZ plus PaZapA, and PaFtsZ plus PaZipA using the light-scattering assay. In HMK buffer (pH 7.5), PaZipA has mild effects on the kinetics of PaFtsZ assembly (Fig. 4A); it is consistent with our EM observation that PaZipA has small effects on the sizes of PaFtsZ filaments (Figs. 1A, 2A). It is a rapid assembly that took around 10 s to the plateau. The weak light-scattering signal is reflected in the single pfs formation. Meanwhile, PaZapA promoted PaFtsZ bundling formation, corresponding to a strong light-scattering signal. For this, we used the reduced slit width in the spectrofluorometer to follow the formation of large bundles. After a short lag bundle assembly occurred slowly and reached a plateau at ~ 200 s (Fig. 4B) and the weak initial formation of single pfs was hidden by the strong fast bundle assembly of PaFtsZ–PaZapA.

In MMK buffer, all of PaFtsZ, PaFtsZ–PaZipA and PaFtsZ–PaZapA assemble into large bundles (Figs. 1D, 2C, 3A), which cause strong light-scattering signals. However, their bundles formation displayed different kinetic properties. PaFtsZ alone showed a slow bundling formation process with a 70 s lag time and around 300 s rising time (Figs. 1H, 4C). On the other hand, the bundling formations were much faster in the presence of PaZipA or PaZapA. PaFtsZ plus PaZipA or PaZapA assembled with very a short lag, reaching a plateau at 50–100 s (Fig. 4C).

There is an obvious contradiction between ZapA inducing FtsZ to form a huge bundle structure and only causing a small change in the GTPase activity of FtsZ. Therefore, we are curious about the effect of ZapA on the polymerization and subunit exchange of FtsZ pfs. Light scattering is strongly affected by the size of the scattering polymers, and therefore measures primarily bundling. We have previously devised fluorescence assays that report assembly of pfs independently of bundling, i.e., the fluorescence signal is directly proportional to the number of subunits in pfs^9,10,46^. Our efforts to engineer the needed constructs in PaFtsZ failed.

The identity between FtsZ from *E. coli* and *P. aeruginosa* is around 62%. Even though both ZipA and ZapA from *E. coli* and *P. aeruginosa* are only less than 30% identical (around 50% positives) (Fig. S1A and B), we found that their effects on the FtsZ pfs, including the sizes, shapes and properties, are very similar. EcZapA also had only a mild effect on the GTPase activity of EcFtsZ, a less than 20% reduction^27,41^. Therefore, we think that the effects and kinetic characteristics of ZapA from *E. coli* and *P. aeruginosa* are similar. Both EcZapA and PaZapA can induce FtsZ assembles into large straight bundles, but both of them have only mild effects on GTP hydrolysis activity of FtsZ. So we turned to EcFtsZ to apply the fluorescence assays to study the effects of ZapA. There are two fluorescence assays we used to measure the kinetics of assembly and subunits turnover of EcFtsZ. One assay uses a bodipy fluorophore was labeled at Cys151 of the double mutant EcFtsZ-T151C/Y222W in the N-terminal subdomain which is quenched by a nearby engineered Trp222 within van der Waals distance in the C-terminal subdomain^24,46^. Upon assembly a conformational change moves the bodipy away from the Trp, increasing its fluorescence. Another FRET (fluorescence resonance energy transfer) assay, which labeled EcFtsZ-L268C with fluorescein as donor and with tetramethylrhodamine as acceptor, can measure initial kinetics of assembly, and also the kinetics of subunit exchange at steady state^10^.

**Figure 5:**
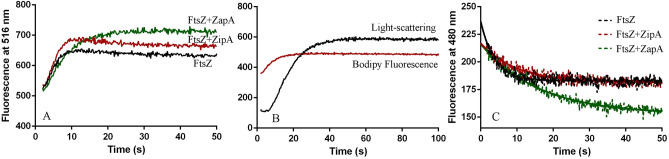
Dynamic properties of EcFtsZ in the absence of or in the presence of EcZipA or EcZapA measured by fluorescence and light-scattering assays. Note that these experiments used EcFtsZ, not PaFtsZ. These experiments were done in HMK buffer. **(A)** Kinetics of EcFtsZ pfs assembly measured by bodipy fluorescence. Assembly of 7 µM EcFtsZ alone is compared with assembly with 10 µM ZapA or ZipA. **(B)** Assembly of 7 µM EcFtsZ plus 10 µM EcZapA measured by bodipy fluorescence, compared to bundling measured by light scattering. **(C)** Exchange of subunits between EcFtsZ pfs was measured by FRET assay, for 7 µM EcFtsZ alone or mixed with 10 µM ZipA or ZapA.

We then used the bodipy assay to see how EcZapA and EcZipA affected the kinetics of EcFtsZ assembly in HMK buffer (Fig. 5A). To minimize the impact of protein labeling, the samples we used contain only less than 10% Bodipy labeled FtsZ, mixed with 90% wild type FtsZ. Assembly of EcFtsZ rose rapidly to a plateau within 10 s, consistent with the rapid assembly of EcFtsZ single pfs. Assembly was almost identical with EcZipA, while EcZapA slowed EcFtsZ assembly somewhat, reaching a plateau in ~ 15 s (Fig. 5A). Figure 5B compares the assembly of EcFtsZ pfs measured by bodipy fluorescence with bundling measured by light scattering for the mixture of EcFtsZ and EcZapA. The bodipy reached a plateau by ~ 15 s, however, the light scattering showed a lag of ~ 10 s, and then rose more slowly to reach a plateau after 30 s. These results suggest that bundling formation might follow the single filaments assembly through the lateral contact of single filaments.

The exchange of subunits at steady state was measured by our FRET assay^10^. For this experiment EcFtsZ-L268C pfs labeled with fluorescein and tetramethylrhodamine were pre-assembled separately and then mixed. The FRET signal, tracked as the decrease in donor fluorescence, measures the exchange of subunits to form mixed pfs. Figure 5C shows that EcFtsZ pfs preassembled with EcZipA exchanged subunits at the same rate as EcFtsZ alone, with halftime around 3.0 ± 1.0 s. EcFtsZ pfs assembled with EcZapA showed four times slower exchange, with halftime about 12.3 ± 1.5 s. Apparently, ZapA induced FtsZ to form huge bundles is consistent with the four times slower of FtsZ pfs subunits exchanges, but there is a contradiction that there is only a small effect on the GTPase activity of FtsZ.

## Discussion

### ZapA induces FtsZ to form straight bundles and PaZipA enhances FtsZ curved conformation

Both ZapA and ZipA are positive regulators of the Z-ring. ZipA is an essential protein for cell division and we failed to knock it out from *P. aeruginosa*. It is reported that some FtsA mutants from *E. coli* may bypass the need of ZipA^42,43^. In contrast, ZapA is not necessary in bacterial division. Similar to the *E.coli* studies, knockout of zapA from *P. aeruginosa* has no obvious effect on bacterial growth and division (Fig. S2).

Previous studies showed both ZipA and ZapA to induce FtsZ to form bundles and thus considered both of them to be Z-ring stabilizers^28,29,37,39^. However, recent studies showed that ZipA from *E. coli* only induced FtsZ bundling when pH below 7^40,41^. Our results here using proteins from *P. aeruginosa* provide further information to confirm these results. PaZapA induced straight PaFtsZ bundling formation in vitro; meanwhile, PaZipA only caused bundling of PaFtsZ at pH 6.5, but not at pH 7.5. This ZipA effect is similar to our previous results with FtsZ and ZipA from *E. coli*^41^. In that study, we further demonstrated that EcZipA promoted and stabilized the FtsZ-GDP highly curved miniring conformation^41^. In the present study of *P. aeruginosa* proteins, we did not find stabilization of minirings in the presence of GTP. Meanwhile, PaZipA enhanced the intermediate curved conformation of PaFtsZ pfs of diameter 50–100 nm. In the previous study of the effects of ZipA from *E. coli*, we mostly focused on the highly curved filaments of diameter 20–30 nm. These highly curved structures appeared mostly at the end of the filament, or sometimes minirings , which is considered as the FtsZ-GDP bending conformation after GTP hydrolysis^41^. We checked it again, and we could also observe many pfs moderately curved, referred to as intermediated curved filaments in the EM images of EcFtsZ-ZipA in our previous published paper^41^. It is consistent with our reports here that ZipA promotes and stabilizes the FtsZ-GTP intermediated curved filaments. The intermediate curved conformation may also be the physiologically important one for forming the ring structures and generating the constriction force^2^. PaFtsZ assembly in HEK buffer gives us a good example of PaZipA also enhancing PaFtsZ assembly: 10 µM PaFtsZ alone is not enough to polymerize; however, 5 µM PaFtsZ plus 10 µM ZipA can assemble into long curved filaments in HEK buffer.

### An apparent contradiction: ZapA bundling hardly affects GTPase, but significantly slows subunit exchange measured by FRET

One might expect that when FtsZ pfs are associated into bundles, the lateral contacts would slow treadmilling and subunit exchange. Indeed this appears to be the case for bundles induced by divalent cations (Ca^2+^ and high concentration Mg^2+^), which reduces significantly the GTPase activity and the recycling of subunits^11,47,48^. However, this seems not to be the case for bundles induced by ZapA. Previous studies showed that ZapA-induced bundles from *B. subtilis* and from *E. coli* only slightly inhibited GTPase activity of FtsZ^29,37^. Our results confirm this for PaFtsZ. Recently, two studies that measured the effect of ZapA on treadmilling of FtsZ, and found that ZapA did not slow the FtsZ treadmilling rate in vitro^49^ and in vivo^50^. Overall these results suggest that the bundling of FtsZ pfs by ZapA is loose enough that it does not significantly alter treadmilling. The interval distance between each filament of PaFtsZ–ZapA bundles is around 5–11 nm from our measurements, close to the previous results of 11–18 nm of EcFtsZ–ZapA^37^.

Nucleotide hydrolysis is generally considered to be a measure of subunit exchange, because once an FtsZ subunit has hydrolyzed its GTP it needs to dissociate from the polymers in order to exchange GDP for GTP and undergo another round of assembly and hydrolysis. One possible explanation is the GTP/GDP exchanges may occur in the middle of filaments. Earlier suggestions that nucleotide might exchange within pfs are discussed in the previous publications^5,51,52^ and are finally contradicted by the structure of pfs of FtsZ from *Staphylococcus aureus*^53,54^. The structure shows that the GDP is trapped in a pocket with no channel that could permit its escape. Now that treadmilling is established as the mechanism for assembly dynamics^14–16,49,50^, we can assume that the rate of GTP hydrolysis at steady state measures the rate at which subunits associate to the bottom and dissociate from the top of pfs.

In contrast to the minimal effect of ZapA bundling on GTP hydrolysis, our FRET assay suggested a fourfold slowing of exchange of fluorescently labeled FtsZ subunits. The subunits exchanges include the cycles of pfs disassembly and re-assembly. The much slower subunits turnover rate is parallel to the longer and larger bundling formation, which is considered to decrease the recycling of subunits. However, it is not proportional to the changes in GTP hydrolysis activity and the rates of treadmilling. During treadmilling process, subunits are bound to one end and release from the other end. This apparent discrepancy between GTPase and FRET is still unclear. It suggests that the rate of GTP hydrolysis determines the rate of treadmilling, but it is not affected significantly by the length of FtsZ pfs, meanwhile, the rate of subunits turnover is related largely to the length of FtsZ filaments.

### Three kinds of FtsZ filaments

This study provides us more evidence about the bending conformation of FtsZ pfs. More and more data supported that there are 3 different kinds of FtsZ pfs: straight, intermediate curved and highly curved^2,41,55–57^. Early reports emphasized that EcFtsZ assembled into mostly straight pfs; however, Erickson and Osawa suggested recently that EcFtsZ pfs actually are a mixture of straight and intermediate curved^2^. These intermediate curved filaments of diameters around 100–300 nm may play important roles in Z-ring formation and force generation^2,5^.

Here we report that PaFtsZ filaments assembled with GTP are mixtures of straight and intermediate curved filaments, and that ZipA induces FtsZ to form more curved pfs. The intermediate curved filaments widely exist across the species. FtsZ from *Caulobacter crescentus* assembles into similar curved filaments^58,59^; Cyanobacterial FtsZ assembles into toroid-like circle bundles of similar curvature^60,61^. Toroids and spiral bundles also were observed when EcFtsZ was assembled in crowding agents^62^, and FtsZ from B. subtilis assembled with GMPCPP or with PC190723, a cell division inhibitor^63^. How straight filaments change to the intermediate curved conformation is still unclear, since it is not coupled with GTP hydrolysis. These two types of filaments often exist together, and a single filament can have a straight and a curved segment.

Another type of curved FtsZ pfs is the highly curved, miniring conformation, with a diameter of 20–30 nm. This highly curved conformation seems to be favored following GTP hydrolysis. In our previous study of EcFtsZ, we observed that the miniring structures could be enhanced and stabilized by ZipA^41^. However, in the present study, PaZipA did not seem to enhance minirings of PaFtsZ with GTP, suggesting that this effect is highly dependent on species. Minirings-like structures were also observed in the cyanobacterial FtsZ pfs^61^.

How the Z-ring generates a constrictive force is still controversial. Clearly, the conformation changes among these three types of filaments may provide the force to bend the membrane. How these bending conformations work together with FtsZ’s treadmilling dynamics in vivo is still unclear. We suggest that the GDP subunits at the minus end of FtsZ filaments switch to a highly curved structure before release, which may produce a continuous contraction force.

## Conclusions

In conclusion, we investigated the unique assembly properties of FtsZ from *P. aeruginosa* and the effects of its two positive regulator proteins, ZipA and ZapA. We found that PaFtsZ pfs are mixtures of straight and intermediate curved filaments. The effects of ZipA on PaFtsZ assembly varied with pH. In physiological conditions, PaFtsZ–ZipA protofilaments were not bundled but promoted more curved filaments. It is consistent with our recent findings in *E. coli* ^41^. It provides us further information that ZipA functions as an enhancer of FtsZ curved filaments during cytokinesis, which may contribute to the force generation to bend the membrane. Comparable to ZapA from other bacterial species, ZapA from *P. aeruginosa* induced PaFtsZ pfs to associate into long straight bundles and/or sheets, which had little effect on the GTPase activity of PaFtsZ. This suggests that the bundles are loose structures and it is consistent with ZapA has little effect on the treadmilling activity of FtsZ filaments^49,50^.

## Methods

### Protein purification

Expression vectors for PaFtsZ, PaZapA, N-terminal truncated PaZipA (26-289), and EcZapA and N-terminal truncated EcZipA (26-328) were constructed in the plasmid pET15b at the NdeI/BamHI sites. The expression vectors were transformed into an *E. coli* strain BL21. Protein expression was induced at 16 °C overnight by the addition of 0.5 mM isopropyl β-d-1-thiogalactopyranoside. After sonication and centrifugation, the soluble His6 proteins were purified by affinity chromatography on a Talon column (Clontech Lab, Inc.). After washing with 0–30 mM imidazole, proteins were eluted with the elution buffer containing 50 mM Tris pH 7.7, 300 mM KCl, 300 mM imidazole.

The purified His6-PaFtsZ was incubated with 2 units/ml of thrombin for 2 h at room temperature to remove the His-tag. A further purification followed by chromatography on a source Q 10/10 column (GE healthcare) with a linear gradient of 50–500 mM KCl in 50 mM Tris, pH 7.9, 1 mM EDTA, 10% glycerol.

*E. coli* FtsZ (EcFtsZ) and mutants (EcFtsZ-F268C, EcFtsZ-T151C/Y222W) were constructed in the plasmid pET11b and were purified as described previously ^10,46^ . Briefly, the soluble bacterially expressed protein was precipitated by 30% saturated ammonium sulfate, followed by chromatography on a source Q 10/10 column (GE healthcare) with a linear gradient of 50–500 mM KCl in 50 mM Tris, pH 7.9, 1 mM EDTA, 10% glycerol. Peak fractions were identified by SDS-PAGE.

The purified proteins were dialyzed into HMK buffer (50 mM HEPES, pH 7.5, 5 mM MgAc, 100 mM KAc), and stored at − 80 °C.

### Buffer used

Most assembly experiments were done in HMK buffer (50 mM HEPES, pH 7.5, 100 mM KAc, 5 mM MgAc) and MMK buffer (50 mM MES, 100 mM KAc, 5 mM MgAc, pH 6.5). Two other buffers, MEK (50 mM MES, 100 mM KAc, 1 mM EDTA, pH 6.5) and HEK (50 mM HEPES, 100 mM KAc, 1 mM EDTA, pH 7.5), were used as noted.

### GTPase activity measurement

GTPase activity was determined by a continuous assay coupled with GTP regeneration system, as described previously^41^. Our assay mixture included 1 mM Phosphoenolpyruvic acid monopotassium (PEP), 0.9 mM NADH, 10 units/ml pyruvate kinase and lactate dehydrogenase (Sigma-Aldrich), and 0.5 mM GTP. In this assay, when GTP is hydrolyzed to GDP, a NADH is consumed in the subsequent reaction, and the GDP in the solution is rapidly regenerated to GTP. The GTP hydrolysis rate is determined through the decrease in absorption of NADH using the extinction coefficient 0.00622 μM^-1^ cm^-1^ at 340 nm. A 3 mm path cuvette was used for measurement. Hydrolysis was plotted as a function of FtsZ concentration, and the slope of the line above the critical concentration (Cc) was taken as the hydrolysis rate. Measurements were made at room temperature with a Shimadzu UV-2401PC spectrophotometer. Each measurement repeats 2 or 3 times.

### Negative stain electron microscopy

Negative stain electron microscopy was used to visualize FtsZ filaments as described previously^41^. Samples of PaFtsZs with or without ZipA or ZapA were incubated with GTP to polymerize for 1–5 min at room temperature. Then, 10 µl samples were applied to a carbon-coated copper grid for about 5 s and then quickly dried with filter papers. Grids were immediately stained with several drops of 2% uranyl acetate. Images were obtained on a Philips EM420 equipped with a CCD camera.

### Light-scattering assay

The assembly kinetics of FtsZ filaments and bundles were measured by light scattering with a Shimadzu RF-5301 PC spectrofluorometer at room temperature. Both excitation and emission were set to 340 nm. Each measurement repeats 2 or 3 times.

### Bodipy fluorescence quenching assay

EcFtsZ assembly kinetics were measured using the Bodipy fluorescence quenching assay as described previously^46^. Bodipy fluorescence can be efficiently quenched by a tryptophan that is close enough to form van der Waals contacts. A double mutant EcFtsZ-T151C/Y222W was labeled with Bodipy-FL *N*-(2-aminoethyl)maleimide (Thermo Fisher Scientific) at Cys 151, and nearby Trp 222 could quench Bodipy fluorescence efficiently. After FtsZ assembly, the bodipy fluorescence increased about 60% due to the conformational changes. Tracking the Bodipy fluorescence changes, we could obtain FtsZ assembly kinetics. For these measurements, the labeled EcFtsZ protein was mixed with a ninefold excess of unlabeled wild-type protein to avoid the changing properties of labeled protein. Bodipy fluorescence was measured at 515 nm, with excitation at 490 nm. Fluorescence measurements were taken with a Shimadzu RF-5301 PC spectrofluorometer at room temperature. Each measurement repeats 2 or 3 times.

### FRET (fluorescence resonance energy transfer) assay

EcFtsZ filaments turn-over rate was measured using a FRET assay, as described previously^10,11^. A single cysteine mutant of EcFtsZ-F268C was labeled separately with Fluorescein 5-maleimide (Thermo Fisher Scientific) as donor and Tetramethylrhodamine 5-maleimide (Thermo Fisher Scientific) as acceptor. FtsZ subunits labeled with each fluorescent dye were mixed with EcZapA (or EcZipA) and polymerized separately for 5 min. The preassembled protofilaments and bundles were then mixed and subunit exchange of FtsZ was tracked using the decrease in donor fluorescence at 515 nm, with excitation at 470 nm. The data were fitted by a single exponential decay, $\mathrm{F}\left(\mathrm{t}\right)=\mathrm{Fo}+\mathrm{a}\times {\mathrm{e}}^{-\mathrm{t}/\uptau }$. Each measurement repeats 2 or 3 times.

## Supplementary Information

Supplementary Figures.

## Abbreviations

PaFtsZ: FtsZ from *Pseudomonas aeruginosa*
PaZipA: ZipA from *Pseudomonas aeruginosa*
PaZapA: ZapA from *Pseudomonas aeruginosa*
EcFtsZ: FtsZ from *Escherichia coli*
EcZipA: ZipA from *Escherichia coli*
EcZapA: ZapA from *Escherichia coli*
pfs: Protofilaments
EM: Electron microscopy
Cc: Critical concentration
FRET: Fluorescence resonance energy transfer
