# Supplementary material for: Assembly properties of bacterial tubulin homolog FtsZ regulated by the positive regulator protein ZipA and ZapA from Pseudomonas aeruginosa
Source: Sci Rep. 2020 Dec 7;10:21369. doi: 10.1038/s41598-020-78431-x (PMC7721900; doi:10.1038/s41598-020-78431-x)
Supplement: Supplementary file 1 — Supplementary Figures. [file 41598_2020_78431_MOESM1_ESM.pdf]

Supplementary Figures

Assembly properties of bacterial tubulin homolog FtsZ regulated by the positive regulator protein ZipA and ZapA from *Pseudomonas aeruginosa*

Mujeeb ur Rahman[1], Zhe Li[1], Tingting Zhang[1], Shuheng Du[1], Xueqin Ma[1], Ping Wang[2], Yaodong Chen[1*]

[1]Key Laboratory of Resources Biology and Biotechnology in Western China, Ministry of Education, College of Life Sciences, Northwest University, Xi'an, Shaanxi, China 710069

[2] Department of Anesthesiology, Duke University Medical Center, Durham, North Carolina USA 27710

* Correspondence to Yaodong Chen: ydchen@nwu.edu.cn

A

```
EcZipA      mmqdlrliliivgaiaiiallvhgfwtsrkerssmf--rdrplkrmkskrdddsydedve      58
PaZipA      mdiglrewlivigliviagilfdgwrrmrggkgklkfkldrsfanl---pdd-----dgd      52
            *  .**  **::* *.* .:*..*:   *  :..:    ** : .:    **     * :

EcZipA      ddegvgevrvhrvnhapanaqeheaarpspqhqyqppyasaqprqpvqqppeaqvppqha      118
PaZipA      saellgparvvehr------------epsfdeqdlpsvsareake-rkg-gkrqeeprq-      97
            . * :* .** . .            .** :.*  *  :: : ::  :   : *  *::

EcZipA      phpaqpvqqpayqpqpeqplqqpvspqvapapqpvhsapqpaqqafqpaepvaapqpepv      178
PaZipA      ---------------gdldldeglalead---------ssdaaetveprkgkskgrkeke      133
                           :  *:: :: :.           . * ::.:* :  :  : *

EcZipA      -aepapvmdkpkrkeaviimnvaahhgselngeallnsiqqagfifgdmniyhrhlspdg      237
PaZipA      rekapavaaepapvdevliinviardesgfkgsallqnilesglrfgdmdifhrhesmag      193
              :   *  :*   : *:*:** *:. * ::*.***:.* ::*: ****:*:*** *  *

EcZipA      sgpalfslanmvkpgtfdpe-mkdfttpgvtifmqvpsygdelqnfklmlqsaqhiadev      296
PaZipA      ngeilfsmanavkpgtfdlddidnfstravsfflglpgprhpkqafdvmvaaarklahel      253
            .*  ***:** ******* : :.:*:* .*::*: :*.  .  * *.:*: :*:::*.*:

EcZipA      ggvvlddqrrmmtpqklreyqdiirevkdana----       328
PaZipA      ngelkdeqrsvltaqtiehyrqriidherrslmqkr       289
            .* : *:** ::* *.:..*:: * : :  .
```

B

```
EcZapA      -msaqpvdiqifgrslrvncppdqrdalnqaaddlnqrlqdlkertrvtnteqlvfiaal      59
PaZapA      msqsntltvqildkeycincpdderanlesaaryldgkmreirssgkvigadrvavmaal      60
              .:: : :**:.:.  :*** *:*  *:.**  *: ::::::.  :* .::::..:***

EcZapA      nisyelaqekaktrdyaasmeqrirmlqqtieqalleqgritektnqnfe  109
PaZapA      nithdllhrkerldqessstrervrelldrvdralanpadagea------  104
            **:::* :.* :  : ::* .:*:* * : :::** : .    *
```

Figure S1. Sequence alignment of ZipA (A) and ZapA (B) from *E. coli* and *P. aeruginosa. ZipA from E. coli and P. aeruginosa are 28% identical (around 45% positive) and ZapA are 24% identical (around 50% positive).*

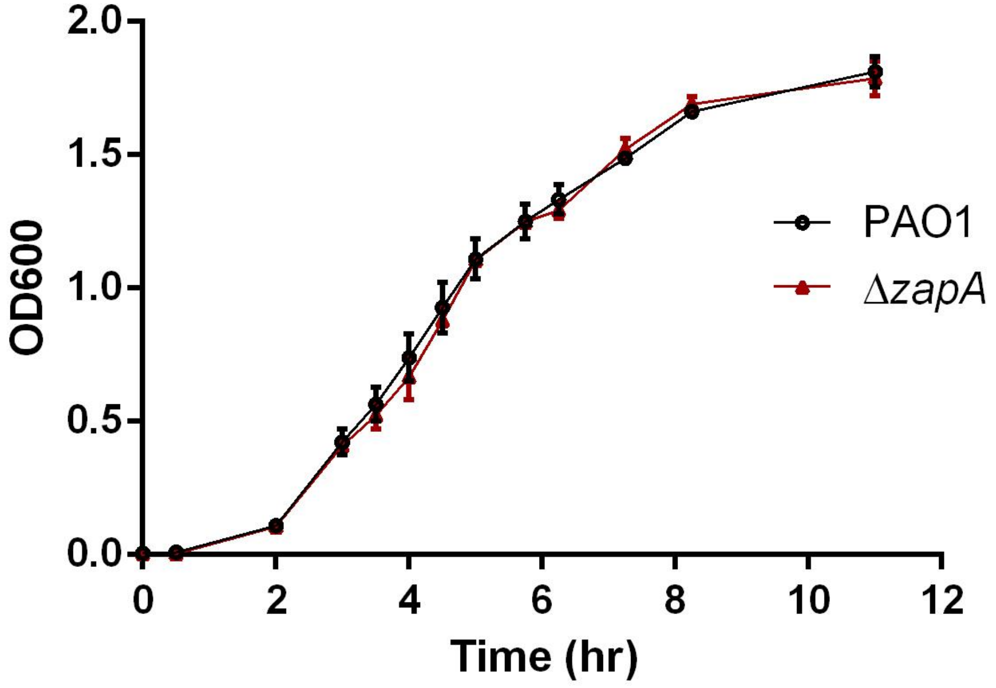

Figure S2. Gene knockout of *zapA* from *Pseudomonas aeruginosa* PAO1 has little effect on the bacterial growth.
